# Factors associated with parental acceptance of the HPV vaccine in girls from metropolitan Lima, Peru

**DOI:** 10.1186/s12889-025-23228-8

**Published:** 2025-07-03

**Authors:** Valeria Juárez-Leon, Delahnie Calderón-Solano, Julio A. Poterico, Jorge Ybaseta-Medina, Diego Azañedo, J. Smith Torres-Román

**Affiliations:** 1 Universidad Científica del Sur, Lima, Peru; 2 Universidad de Huánuco, Huánuco, Peru; 3 Hospital Nacional Docente Madre Niño San Bartolomé, Lima, Peru; 4 Universidad Nacional San Luis Gonzaga de Ica, Ica, Peru

**Keywords:** Cervical cancer, HPV vaccine, Human papillomavirus, Parental acceptance, Peru

## Abstract

**Background:**

In 2022, cervical cancer was the second most common neoplasia among women in Peru, with 4809 cases and 2545 deaths reported. Infection with human papillomavirus (HPV) is a key factor in the development of this disease. Vaccination is the primary strategy for HPV prevention. Although HPV vaccination coverage has improved, evaluating acceptance profiles is crucial for effectively monitoring public immunization policies. We aimed to determine the associated factors with parental acceptance of the HPV vaccine in girls from Peru.

**Methods:**

An observational, analytical, and cross-sectional study was conducted with a sample of 204 parents of girls and adolescents aged 9 to 19 years in Metropolitan Lima. The dependent variable was acceptance of the HPV vaccine, while the independent variables included demographic data, level of knowledge about HPV, and socioeconomic and cultural factors.

**Results:**

A total of 204 parents participated in the study. Overall, 61.3% expressed willingness to vaccinate their daughters against HPV. Factors significantly associated with vaccine acceptance in the bivariate analysis included being a mother, being married, residing in the South-Central region, having a high monthly income, possessing private or public health insurance, and having a higher level of education. In both bivariate and multivariable analyses, having a daughter previously vaccinated against HPV was significantly associated with HPV vaccine acceptance, and remained the only independent predictor in the adjusted model (aPR = 1.25, 95% CI: 1.03–1.53, *p* = 0.023). Knowledge level about HPV was not significantly associated with vaccine acceptance in the adjusted model.

**Conclusion:**

Prior experience with HPV vaccination was the strongest predictor of parental acceptance. Increasing awareness and addressing misconceptions through educational campaigns may enhance vaccine uptake.

## Introduction

Globally, Human Papillomavirus (HPV) is recognized as the most common viral infection of the reproductive tract, representing a significant public health concern due to its relation to specific cancers [1]. Key risk factors associated with HPV infection include having multiple sexual partners, early initiation of sexual activity, inconsistent condom use, oral sex, alcohol and cigarette consumption [2].

In 2012, cervical cancer was the fourth most prevalent cancer and the fourth leading cause of cancer-related mortality among women globally, responsible for approximately 265,700 deaths. This statistic underscored the significance of cervical cancer as a pressing global health challenge, especially in less developed settings [3]. However, by 2022, data from GLOBOCAN indicated that cervical cancer had fallen to the fourth most common cancer among women worldwide, with662 301 new cases (6.9% of the total) and 348 874 deaths (8.1% of the total) reported [4]. Although mortality rates for cervical cancer have declined in many high-income countries in recent years, the burden remains disproportionately high in low- and middle-income nations [2, 5–8]. For instance, cervical cancer reemerged as one of the most prevalent neoplasia among Peruvian women in 2022, representing 12.3% of all cancer cases in this population [9]. Alarmingly, the Amazon region reports particularly high mortality rates associated with this disease [10]. Although the HPV vaccine was incorporated into the national immunization schedule for girls aged 9 to 13 in 2011 by the Peruvian Ministry of Health [11], the full population-level impact on cervical cancer mortality is not yet evident due to the long latency period between HPV infection and disease onset.

In recent years, there has been a significant effort to achieve high coverage rates of HPV vaccinations in Peru, particularly among school-aged children [11]. For instance, in 2023, the Ministry of Health (MINSA) administered 688,489 doses of the human papillomavirus (HPV) vaccine to boys and girls aged 9 to 13, achieving 87% coverage of the target population and contributing to a reduction in cervical cancer incidence. Notably, regions such as Lima and several others achieved 100% coverage, while some limited-resource areas of the country still face challenges in reaching this goal [12].

To maintain high vaccination coverage rates against HPV and prevent cancers associated with these infectious agents, it is essential for both parents and children to understand the importance of vaccines and their benefits, not only for their individual health but also for public health overall. Parents play a crucial role in this context, as they are responsible for providing consent for the vaccination of their daughters. Numerous studies in high-income settings have explored the factors influencing parental acceptance of the HPV vaccine [13–15]. However, there remains a lack of context-specific evidence from low- and middle-income countries, particularly in urban Peru, where cultural norms, health system structure, and socioeconomic conditions may shape parental decision-making differently.

Investigating the factors that may influence the acceptance of the HPV vaccine among Peruvian children is essential for improving vaccination rates. This research will enable the implementation of timely measures that enhance the prevention of cervical cancer and other cancers associated with HPV. Therefore, this study aims to identify the factors associated with parental acceptance of the HPV vaccine for their daughters in Metropolitan Lima, main zone of the capital Lima in Peru.

## Materials and methods

### Data source

This cross-sectional analytical observational study was conducted in Metropolitan Lima, the capital of Peru, which comprises 43 urban districts and represents the most densely populated region of the country [16]. Although data collection was conducted virtually, participation was restricted to residents of Metropolitan Lima. This was ensured by including a mandatory question on district of residence in the survey form. Respondents who indicated residence outside Metropolitan Lima were excluded during data cleaning.

The target population consisted of parents or legal guardians of girls aged 9 to 19 years. Although the HPV vaccine is currently recommended in Peru for both girls and boys aged 9 to 13 years, this study focused specifically on parents of girls for two key reasons. First, since its introduction in the Peruvian national immunization schedule in 2011, HPV vaccination has primarily targeted girls, with school-based campaigns and communication strategies historically focused on the prevention of cervical cancer. As a result, parental attitudes toward vaccinating daughters remain particularly relevant to national HPV prevention efforts. Second, the selected age range of 9 to 19 years was intended to include not only girls within the current vaccination target group (9–13 years) but also older adolescents who may have missed earlier doses and are still eligible for catch-up vaccination under Ministry of Health guidelines. This age range also aligns with the window during which most parents make vaccination decisions for their daughters.

Only parents who resided in Metropolitan Lima, had at least one daughter aged 9 to 19 years, and provided informed consent were included. Exclusion criteria included incomplete surveys and responses from individuals who did not meet the eligibility requirements or failed to provide valid consent.

The sample size was determined using EPIDAT 4.2, based on a previous local study that reported 78.3% of parents had vaccinated their children against HPV [17]. Since the total population of eligible parents was unknown, the sample size was determined using the formula for infinite populations. Assuming a 95% confidence level, the minimum required sample size for this study was estimated to be 222 parents.

### Variables and data collection instrument

The dependent variable for this study was parental acceptance of the HPV vaccine, categorized as “would accept” or “would not accept” vaccination for their daughters. The independent variables encompassed demographic information (relationship to the child, marital status, and place of residence), knowledge about HPV, socioeconomic factors (monthly income, type of health insurance, educational level, occupation, and number of children), and cultural factors (religion, prior HPV vaccination of the daughter, and history of cervical cancer).

The primary instrument utilized was a questionnaire developed based on the study variables and previous research [18, 19]. The knowledge section of the questionnaire was adapted from a study conducted by Villalobos Guillermo [20], which assessed knowledge and attitudes toward the HPV vaccine among mothers of primary school students in urban Arequipa. The instrument underwent content validation by a panel of experts comprising three gynecology-obstetrics specialists from Hospital III Goyeneche in Arequipa, a registered nurse responsible for vaccination strategy at the La Joya Health Network, and a social worker from the same network. The instrument exhibited strong reliability, achieving a Cronbach’s alpha coefficient of 0.78.

The questionnaire comprised a total of 21 questions. The first question determined whether participants had a daughter aged 9 to 19 years. If the answer was affirmative, participants proceeded to complete the remaining 20 questions, organized into five sections: demographic information (4 questions), knowledge about HPV (7 questions), acceptance of the HPV vaccine (1 question), socioeconomic information (5 questions), and cultural factors (3 questions). Knowledge about HPV vaccination was assessed using seven multiple-choice questions, each with a single correct answer. Participants received one point for each correct answer, and their total score ranged from 0 to 7. This score was then categorized into three fixed tertiles to classify knowledge levels as follows: low knowledge (0–2 points), moderate knowledge (3–5 points), and high knowledge (6–7 points). These categories were used as an ordinal variable in both descriptive and inferential analyses.

The knowledge section aimed to evaluate participants’ cognitive understanding of HPV through questions about diseases caused by the Human Papillomavirus (HPV), modes of transmission, purpose of the vaccine, target population for vaccination, recommended age for administration, method and dosage schedule, optimal timing for vaccination, and potential side effects of the vaccine. A database was created using a questionnaire in Google Forms, which was distributed via social media platforms such as Facebook, Twitter, and Instagram. Data were collected virtually using non-probabilistic convenience sampling techniques, and a segmentation algorithm was employed to optimize dissemination.

The segmentation algorithm focused on identifying specific user groups within social media platforms and targeting them more effectively with the research questionnaire. An analysis of the target audience was conducted, considering interests aligned with our study topic. Subsequently, segmentation tools provided by social media platforms, such as custom audience creation and interest-based targeting, were utilized to divide and direct our outreach to individuals likely to be interested in the subject matter. This approach allowed us to tailor our messaging and include the questionnaire link in a manner that was relevant and engaging for this audience, enhancing the likelihood of participation and ensuring more accurate data collection. Additionally, we identified online communities with shared interests related to our topic and provided them with the link to our form.

### Statistical analysis

The dataset was initially recorded in Excel and subsequently imported into IBM SPSS Statistics 25 for analysis. Qualitative variables were summarized using absolute (n) and relative frequencies (%), while quantitative variables were described using means and standard deviations (SD). A bivariate analysis was performed to evaluate the association between the independent variables and the dependent variable (acceptance of the HPV vaccine). Student’s t-test for independent samples was used for age and the dependent variable. For the comparison of two categorical variables, chi-square (expected frequencies in the cells were ≥ 5) or Fisher’s test (expected frequencies < 5) was used.

For the multivariate analysis, Poisson regression was employed, given that the dependent variable was dichotomous and the aim was to predict its behavior based on other influencing factors. The association was expressed as prevalence ratios (PR), presented with both crude and adjusted values, accompanied by 95% confidence intervals (95% CI). Variables included in the adjusted model were selected based on statistical significance, specifically those demonstrating a *p*-value of less than 0.05 in the bivariate analyses.

### Ethical aspects

Participants were clearly informed, in plain language, about the purpose of the study, the guarantees of confidentiality and anonymity, and the researchers’ contact information. A detailed explanation of the study’s objectives was provided, and participants were informed of their right to refrain from answering the questionnaire without facing any negative consequences. This study received approval from the Ethics Committee of Universidad Cientifica del Sur (CONSTANCIA N°313-CIEI-CIENTIFICA-2022). All participants provided informed consent before taking part in the study. The research was conducted in accordance with the principles outlined in the Declaration of Helsinki and complied with all relevant national ethical regulations for research involving human subjects. Additionally, no conflicts of interest were reported among the researchers responsible for this project. All participants were able to read and understand the informed consent. No cases of illiteracy or incapacity to consent were reported or identified during the study.

## Results

A total of 235 responses were obtained from parents; however, after excluding 31 participants who did not meet the eligibility criteria, the final sample was made up of 204 parents.

The majority of respondents were mothers (*n* = 155, 76.0%). In terms of marital status, cohabitation with a partner was the most common situation reported (*n* = 90, 44.1%). Additionally, most respondents resided in the North region of Lima (*n* = 60, 29.4%). Regarding knowledge of HPV vaccination, it was observed that most parents (*n* = 93, 45.6%) exhibited a medium level of knowledge, while 63 participants (30.9%) demonstrated a high level of knowledge about HPV vaccination. In terms of socioeconomic factors, 69.1% of participants (*n* = 141) reported having a high monthly income (above 2,400 soles), and 75% (*n* = 153) had public health insurance. Over 33.3% (*n* = 68) of respondents had completed university education. At the time of the survey, 78.9% (*n* = 161) were employed, and a majority reported having two children (*n* = 96, 47.1%). Furthermore, 72.5% (*n* = 148) identified as Catholic. Interestingly, despite 43 participants (21.1%) reporting a family history of cervical cancer, most had not vaccinated their daughters against HPV (*n* = 135, 66.2%). Conversely, a significant number of participants (*n* = 125, 61.3%) expressed their HPV vaccine acceptance their daughters (Table 1).

**Table 1:** Demographic, socioeconomic, cultural, and knowledge characteristics about HPV vaccination among parents residing in metropolitan Lima - Peru (*n* = 204)

Variables	*n*	%
**Age (mean) ± SD**	38.5 ± 6.2	
**Relationship with the girl or adolescent**		
Father	49	24.0
Mother	155	76.0
**Marital status**		
Married	44	21.6
Cohabiting	90	44.1
Divorced/separated/Widowed	49	24.0
Single	21	10.3
**Residence**		
South	41	20.1
East	38	18.6
Central	27	13.2
South-Central	38	18.6
North	60	29.4
**Level of knowledge about HPV vaccination**		
Low	48	23.5
Medium	93	45.6
High	63	30.9
**Monthly income [n (%)]**		
Low (less than 900 soles)	11	5.4
Medium (900–2400 soles)	52	25.5
High (more than 2400 soles)	141	69.1
**Type of health insurance**		
None	10	4.9
Public	153	75.0
Private	41	20.1
**Level of education**		
No studies/Primary school completed	15	7.4
High school completed	51	25.0
Technical education	70	34.3
University education	68	33.3
**Occupation**		
Employed	161	78.9
Unemployed	43	21.1
**Number of children**		
1	62	30.4
2	96	47.1
≥ 3	46	22.5
**Religion**		
None	12	5.9
Catholic	148	72.5
Evangelical	31	15.2
Other	13	6.4
**Have you vaccinated your daughter against HPV?**		
No	135	66.2
Yes	69	33.8
**Family history of cervical cancer**		
No	161	78.9
Yes	43	21.1
**Would you vaccinate your daughter against HPV?**		
No	79	38.7
Yes	125	61.3

Parental willingness to vaccinate their daughters varied significantly across several demographic and socioeconomic factors. Mothers (65.1%) were more likely to vaccinate than fathers (48.9%) (*p* = 0.04). Similarly, married (84.1%) and divorced/separated/widowed parents (75.5%) showed higher willingness compared to cohabiting parents (40.0%) (*p* < 0.001). The parents from the South-Central region (89.5%) having the highest acceptance rates, while those from the North (45.0%) showed the lowest (*p* < 0.001). Socioeconomic factors also played a crucial role. High-income parents (74.5%) were significantly more willing to vaccinate compared to low-income parents (27.3%) (*p* < 0.001). Health insurance status influenced vaccination acceptance, with private (78.1%) and public insurance holders (60.1%) showing greater influenced than uninsured parents (10.0%) (*p* < 0.001). Higher education levels were strongly associated with vaccine acceptance, as 97.1% of university-educated parents expressed willingness compared to 20.0% of those with no formal education (*p* < 0.001). Employment was another key factor, with employed parents (70.2%) more likely to vaccinate than unemployed parents (27.9%) (*p* < 0.001). Prior vaccination experience was the strongest predictor of vaccine acceptance; 94.2% of parents whose daughters had already been vaccinated reported willingness to continue, compared to 44.4% of those whose daughters had not been vaccinated (*p* < 0.001). Additionally, higher knowledge about HPV vaccination was significantly associated with increased vaccine acceptance (*p* = 0.001). In contrast, religion was not a significant factor influencing vaccination decisions (*p* = 0.53) (Table 2).

**Table 2:** Association between demographic, socioeconomic, cultural factors, and knowledge about HPV vaccination with parents’ willingness to vaccinate their daughters in metropolitan Lima, Peru (*n* = 204)

Variables	No (*n* = 79)	%	Yes (*n* = 125)	%	*p*-value
**Demographic Factors**					
**Age (mean) ± SD**	36.9 ± 5.4		39.5 ± 6.4		0.004
**Relationship with the girl or adolescent**					
Father	25	51.2	24	48.9	0.04
Mother	54	34.8	101	65.1	
**Marital status**					
Married	7	15.9	37	84.1	*p* < 0.001
Cohabiting	54	60.0	36	40.0	
Divorced/separated/Widowed	12	24.5	37	75.5	
Single	6	28.6	15	71.4	
**Residence**					
South	19	46.3	22	53.7	*p* < 0.001
East	13	34.2	25	65.8	
Central	10	37.0	17	62.9	
South-Central	4	10.5	34	89.5	
North	33	55.0	27	45.0	
**Socioeconomic Factors**					
**Monthly income**					
Low (< 900 soles)	8	72.7	3	27.3	*p* < 0.001
Medium (900–2400 soles)	35	67.3	17	32.7	
High (> 2400 soles)	36	25.5	105	74.5	
**Health insurance type**					
None	9	90.0	1	10.0	*p* < 0.001
Public	61	39.9	92	60.1	
Private	9	21.9	32	78.1	
**Education level**					
No education/Primary school	12	80.0	3	20.0	*p* < 0.001
High school	35	68.6	16	31.4	
Technical education	30	42.9	40	57.1	
University education	2	2.9	66	97.1	
**Occupation**					
Employed	48	29.8	113	70.2	*p* < 0.001
Unemployed	31	72.1	12	27.9	
**Number of children**					
1	22	35.5	40	64.5	
2	36	37.5	60	62.5	
≥ 3	21	45.6	25	54.4	
**Cultural Factors**					
**Religion**					
None	6	50.0	6	50.0	0.535
Catholic	55	37.2	93	62.8	
Evangelical	11	35.5	20	64.5	
Other	7	53.9	6	46.2	
**Has vaccinated daughter against HPV?**					
No	75	55.6	60	44.4	*p* < 0.001
Yes	4	5.8	65	94.2	
**Family history of cervical cancer**					
No	70	43.5	91	56.5	0.007
Yes	9	20.9	34	79.1	
**Knowledge level about HPV vaccination**					
Low	29	60.4	19	39.6	0.001
Medium	33	35.5	60	64.5	
High	17	26.9	46	73.0	

Although bivariate analysis showed a significant association between knowledge level and vaccine acceptance (*p* = 0.01), this association was not maintained in the multivariate model (high knowledge: aPR = 0.98, 95% CI: 0.70–1.38, *p* = 0.92). Having a daughter previously vaccinated against HPV was the strongest predictor of vaccine acceptance (aPR = 1.25, 95% CI: 1.03–1.53, *p* = 0.02). In contrast, factors such as parental age, marital status, residence, income, health insurance, education level, and family history of cervical cancer lost statistical significance. Religion and the number of children were not associated with vaccine acceptance in either the crude or adjusted model (Table 3).

**Table 3:** Crude and adjusted multivariate analysis of parents’ willingness to accept HPV vaccination for their daughters in metropolitan Lima (*n* = 204)

Variable	Crude PR	95% CI	*p*-value	Adjusted PR	95% CI	*p*-value
**Age**	1.03	1.01–1.04	0.001	1.01	0.99–1.02	0.205
**Relationship with the girl or adolescent**						
Father	**Ref**					
Mother	1.33	0.98–1.81	0.070			
**Marital Status**						
Married	**Ref**			**Ref**		
Cohabiting	0.48	0.38–0.63	< 0.001	0.75	0.56–1.01	0.061
Divorced/Separated/Widowed	0.90	0.73–1.10	0.304	1.03	0.85–1.26	0.741
Single	0.85	0.63–1.15	0.287	0.89	0.70–1.13	0.345
**Residence**						
Central	**Ref**			**Ref**		
East	1.04	0.72–1.51	0.816	1.00	0.74–1.35	0.986
South-Central	1.42	1.04–1.94	0.026	1.22	0.94–1.57	0.131
North	0.71	0.48–1.07	0.103	0.96	0.69–1.33	0.784
South	0.85	0.57–1.28	0.441	1.02	0.71–1.47	0.910
**Monthly Income**						
Low	**Ref**			**Ref**		
Middle	1.20	0.42–3.40	0.734	0.84	0.36–1.94	0.680
High	2.73	1.03–7.22	0.043	1.25	0.55–2.83	0.591
**Health Insurance Type**						
None	**Ref**			**Ref**		
Public	6.01	0.93–38.95	0.060	3.71	0.56–24.69	0.174
Private	7.80	1.20–50.69	0.031	3.52	0.53–23.41	0.193
**Education Level**						
No studies/Primary school completed	**Ref**			**Ref**		
High school completed	1.57	0.53–4.68	0.420	0.85	0.30–2.47	0.763
Technical education	2.86	1.02–8.04	0.047	1.26	0.44–3.57	0.667
University education	4.85	1.76–13.40	0.002	1.51	0.53–4.24	0.439
**Occupation**						
Unemployed	**Ref**					
Employed	2.52	1.54–4.11	< 0.001			
**Number of children**						
1	**Ref**					
2	0.97	0.76–1.23	0.797			
≥ 3	0.84	0.61–1.16	0.299			
**Religion**						
None	**Ref**					
Catholic	1.26	0.70–2.25	0.440			
Evangelical	1.29	0.69–2.41	0.424			
Other	0.92	0.41–2.09	0.848			
**Vaccinated Daughter Against HPV?**						
No	**Ref**			**Ref**		
Yes	2.12	1.74–2.58	< 0.001	1.25	1.03–1.52	0.021
**Family History of Cervical Cancer**						
No	**Ref**			**Ref**		
Yes	1.40	1.14–1.72	0.001	1.11	0.94–1.31	0.224
**Knowledge level about HPV vaccination**						
Low	**Ref**			**Ref**		
Medium	1.63	1.11–2.39	0.012	1.04	0.73–1.47	0.844
High	1.84	1.26–2.70	0.002	0.98	0.70–1.38	0.921

## Discussion

This study provides important insights into the factors associated with parental acceptance of the HPV vaccine for their daughters in Metropolitan Lima. Our findings indicate that prior vaccination experience was the strongest predictor of willingness to vaccinate, reinforcing the role of familiarity and trust in immunization programs. While socioeconomic factors such as income, education level, and health insurance were initially associated with vaccine acceptance, these associations lost statistical significance in the multivariate model, suggesting that other underlying factors may play a more decisive role. Additionally, although a higher level of knowledge about HPV was linked to greater acceptance in the bivariate analysis, this association was not maintained after adjusting for other variables, indicating that knowledge alone may not be sufficient to drive vaccination decisions. These results highlight the complexity of parental attitudes toward HPV vaccination and underscore the need for comprehensive strategies that go beyond knowledge dissemination to address hesitancy and accessibility barriers.

In this study, 33.8% of parents reported having vaccinated their daughters against HPV, a figure that highlights both progress and gaps in vaccine uptake. This percentage is lower than HPV vaccination coverage reported in some high-income countries [21], where national immunization programs have achieved rates above 70–80% through school-based initiatives and widespread public health campaigns. This low HPV vaccination coverage both in Peru and in other countries could be mainly due to parents’ refusal to have their daughters vaccinated, due to lack of information, lack of knowledge about the disease, fear of side effects and uncertainty about vaccine effectiveness, as well as cost and lack of healthcare [22–25].

In our research, 61.3% of parents in Metropolitan Lima expressed their willingness to vaccinate their daughters against HPV. Among these, married parents were more likely to accept vaccination compared to cohabiting parents. This distinction can be interpreted as reflecting a formalized commitment rather than reinforcing gender stereotypes or assumptions about specific roles within marriage. This finding is consistent with Oliveira A.‘s research in Peru, which showed that 87.5% of married parents accepted HPV vaccination for their daughters [17]; however, no statistical significant association was found between marital status and willingness to vaccinate. Similarly, Chaupis-Zevallos reported no such association, though in that case, cohabiting parents (51.5%) were more likely to vaccinate their daughters than married parents (34.5%) [18].

Previous studies in Spain and Peru have identified fear of potential side effects as the primary reason for parental hesitancy regarding HPV vaccination [26, 27]. However, positive recommendations from healthcare professionals have been shown to enhance vaccination acceptance when these professionals actively advocate for the vaccine [26]. This underscores the importance of effective communication from healthcare providers to address concerns and mitigate fears surrounding potential side effects.

Our study also indicated that having a university education was positively associated with the acceptance of HPV vaccination for daughters, aligning with Oliveira A.‘s findings that acceptance was linked to having technical or higher education [17]. Educational attainment proved to be a critical factor in vaccine acceptance, corroborated by studies conducted by Chaupis-Zevallos (Peru), Millán-Morales (Mexico), and Jurado C. (Argentina) [18, 28, 29]. These studies uniformly demonstrated a direct relationship between higher academic achievement and parental willingness to vaccinate their daughters against HPV, aligning with our findings. Thus, it can be asserted that greater knowledge correlates with higher vaccine acceptance. However, while these associations were significant in the bivariate analysis, they did not remain significant in the multivariate model, suggesting that their effects may be confounded or mediated by other variables, such as pre-vaccination exposure. Although these results point to possible trends, they should be interpreted with caution and not as independent predictors of vaccine uptake.

The findings of this study highlight the need for targeted public health interventions to improve HPV vaccine acceptance among parents in Peru. The finding that having a daughter previously vaccinated against HPV was the strongest predictor of vaccine acceptance, this underscores the importance of prior vaccination experiences in shaping parental attitudes toward immunization. This aligns with previous studies indicating that parents who have already engaged with HPV vaccination programs exhibit higher trust in vaccine safety and effectiveness, making them more likely to support additional doses or recommend vaccination to others [30, 31]. Policymakers should focus on increasing initial uptake through school-based immunization programs and community outreach initiatives. Additionally, our results suggest that parental knowledge alone does not necessarily translate into higher acceptance rates, emphasizing the importance of combining educational efforts with trust-building strategies. Health authorities should implement communication campaigns led by healthcare professionals to address vaccine hesitancy, particularly in lower-income and less-educated populations. Furthermore, expanding access to vaccination through mobile clinics and partnerships with private healthcare providers may help reach underserved communities and increase overall coverage.

It is important to interpret the study findings in light of potential selection bias. Because data collection was conducted virtually and relied on convenience sampling, the sample likely overrepresents individuals with better internet access, higher digital literacy, and greater interest in health topics. As such, parents from lower socioeconomic backgrounds or those with limited access to online platforms may be underrepresented. This may have skewed the overall rates of HPV vaccine acceptance upward and limits the generalizability of the findings to the broader population of Metropolitan Lima. Future studies using random sampling methods and mixed-mode data collection (including in-person surveys) are recommended to obtain more representative data and confirm these associations across diverse subgroups.

### Limitations and strengths

This study has several limitations. First, its cross-sectional design restricts the ability to establish causal relationships, as it only identifies statistical associations between parental willingness to vaccinate their daughters and the studied variables. Future prospective and longitudinal studies are needed to determine whether these associations represent true causal effects. Second, data collection was conducted virtually during the COVID-19 pandemic, raising concerns about self-reporting bias and the accuracy of participant responses. Although measures were implemented to enhance data validity—clearly outlined in the informed consent and survey instructions—the lack of direct verification mechanisms may have introduced response bias, affecting the generalizability of findings.

Another limitation of this study is that the sample size was estimated to determine a proportion rather than to assess associations between variables. As a result, the findings should be interpreted as exploratory rather than definitive. The lack of significant associations for certain variables in the multivariate analysis may be attributed to the small sample size within some categories, limiting the statistical power to detect true relationships. Future studies with larger and more representative samples are needed to confirm these findings and provide a more comprehensive understanding of the factors influencing parental acceptance of the HPV vaccine. Other limitations include that data collection was conducted virtually, this study is subject to potential selection biases that may limit the generalizability of findings. First, self-selection bias is likely, as individuals with greater interest in health-related topics or more favorable attitudes toward vaccination may have been more inclined to participate. Second, the online format may have introduced digital access and literacy bias, disproportionately excluding individuals with limited internet access or lower digital skills, such as older adults or members of low-income households. These factors may have resulted in the underrepresentation of vulnerable or marginalized populations. Although measures were taken to minimize these issues—such as screening responses for inconsistencies and reinforcing the informed consent process—these limitations may have affected data quality and should be considered when interpreting the study results. On the other hand, this study has several strengths. First, it provides valuable insights into parental acceptance of the HPV vaccine in Peru, a topic with limited prior research in the country. By including a diverse sample of parents from different socioeconomic backgrounds within Metropolitan Lima, the study captures a broad range of perspectives and factors influencing vaccine acceptance. Second, the use of a validated questionnaire with strong reliability ensures the accuracy and consistency of the collected data. Third, the study employs a robust statistical approach, including both bivariate and multivariate analyses, to identify key predictors of vaccine acceptance. These findings can serve as a foundation for future research and inform public health strategies aimed at increasing HPV vaccination rates. Lastly, the study highlights the importance of prior vaccination experiences in shaping parental attitudes, which can guide targeted interventions to enhance vaccine coverage.

## Conclusion

In conclusion, this study identified prior HPV vaccination of a daughter as the strongest independent factor associated with parental acceptance of the HPV vaccine. These findings highlight the importance of prior exposure to HPV vaccination in shaping positive attitudes toward immunization. However, given the use of virtual data collection and non-probabilistic sampling, the results may not be generalizable to all parents in Metropolitan Lima or other regions of Peru. Future research with more representative sampling methods is needed to validate and extend these findings.

## Data Availability

Availability of data and materialsThe datasets generated and/or analysed during the current study are available are available upon request.
